# Spatially resolved readout of a Fabry–Perot ultrasound sensor interrogated through a multimode optical fiber using wavefront shaping

**DOI:** 10.1063/5.0166826

**Published:** 2023-11-17

**Authors:** Benjamin Keenlyside, Dylan Marques, Nathaniel Redgewell, Maxim Cherkashin, Edward Zhang, Paul Beard, James Guggenheim

**Affiliations:** 1 Department of Medical Physics and Biomedical Engineering, University College London, London, United Kingdom; 2 Institute of Cardiovascular Sciences, University of Birmingham, Birmingham, United Kingdom; 3 School of Engineering, University of Birmingham, Birmingham, United Kingdom

## Abstract

The spatially resolved interrogation of a Fabry–Perot ultrasound sensor using a laser beam focused through a multimode fiber is demonstrated. To scan the beam across the sensor as required to read it out, optical wavefront shaping was employed to compensate for the scrambling of light in the fiber. By providing a means to map ultrasound through inexpensive, lightweight fibers, this could lead to new ultrasonic and photoacoustic imaging systems, such as endoscopes and flexible handheld probes.

Fabry–Perot (FP) ultrasound sensors (FPUSs)^1^ are a class of all-optical ultrasound sensors based on compressible optical microcavities. A typical FPUS comprises an optically clear layer between two planar mirrors. When illuminated by a focused laser beam, this forms an interferometer, whose transmissivity varies with wavelength according to an “interferometer transfer function” (ITF). The ITF contains sharp resonances centered on specific wavelengths determined by the thickness of the FPUS. Ultrasonic waves modulate the thickness, shifting the resonances. As such, illuminating the FPUS at a “bias” wavelength at the edge of a resonance and monitoring the reflected power allows measuring ultrasonic waves at the location of the readout beam.

Applying this measurement process at multiple locations by scanning a readout beam across an FPUS allows broadband ultrasonic mapping with microscale spatial sampling and high (e.g., >100 000^2^) channel counts. This has enabled FPUS systems to provide high fidelity, high-resolution ultrasonic images in areas including tomography-mode photoacoustic imaging,^1,3,4^ ultrasonic field characterization,^5^ and pulse-echo^6^ imaging.

While systems typically use free-space optics with rotating mirrors to scan the beam, here we demonstrate an alternative readout concept, shown in Fig. 1. Briefly, a focused beam is scanned across an FPUS by delivering structured light through a multi-mode optical fiber (MMF).

**FIG. 1.**
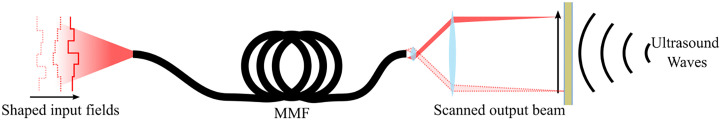
Concept of mapping US fields by interrogating an FPUS using WS through MMF. A shaped input field is coupled into the fiber, chosen such that it emerges as a beam focused on the FPUS. By directing this focus to different positions on the FPUS, an ultrasonic field can be mapped.

Imaging through MMF has recently gained significant attention in other areas including wide field optical,^7^ fluorescence,^8^ 3D time-of-flight,^9^ and multi-modality endomicroscopy.^10^ One attraction of MMF is that it can be hair-thin, with diameters down to 100 *μ*m, while retaining the high information-carrying capacity required to enable multi-channel measurements. Other significant attractions include that MMF is affordable, lightweight, flexible, and tolerant to extreme conditions, such as strong electromagnetic fields^11^ and radioactive environments.^12^ Exploiting these features, reading out an FPUS through MMF could enable creating flexible, handheld, environmentally resilient ultrasonic imaging devices, such as photoacoustic endoscopes,^13^ or flexible, hand-held ultrasound probes.

Despite the attractions, scanning beams through MMF present a challenge. Due to modal dispersion in the MMF, conventional laser beams transmitted into MMF tend to emerge as pseudo-random speckle, bearing no resemblance to the incident beam. This makes it impossible to deliver conventional focused beams through MMF by scanning a beam across the proximal fiber facet, complicating imaging, and sensor readout. Fortunately, the significant recent interest in MMF based endomicroscopy has sparked the emergence of a class techniques affording possible solutions to this challenge. These techniques are based on wavefront shaping (WFS),^14^ the basis of which is that, although light propagation in MMF produces seemingly random speckle, the transmission is nevertheless linear and deterministic. As such, given any physically realizable output field, it is possible, in principle, to determine an appropriate incident wavefront that creates this field at the output of the MMF. Once known, such wavefronts can be generated using a spatial light modulator (SLM). This enables scanning focused beams through MMF. Moreover, different ways of obtaining the required wavefront have now emerged including iterative WFS,^14^ direct transmission matrix (TM) measurement,^15^ and phase conjugation^16^ methods. Here, we apply an iterative genetic algorithm (GA)^17^ to enable reading out an FPUS, thereby making spatially resolved ultrasound measurements, through an MMF.

A schematic of the setup is shown in Fig. 2. Briefly, a beam from a wavelength tunable laser was expanded and filtered to illuminate a region of an SLM. The light reflected from the SLM was imaged onto the tip of an MMF. Light exiting the MMF was delivered via a beam splitter to both (i) an FPUS and (ii) a co-planar camera effectively imaging the illumination on the FPUS.

**FIG. 2.**
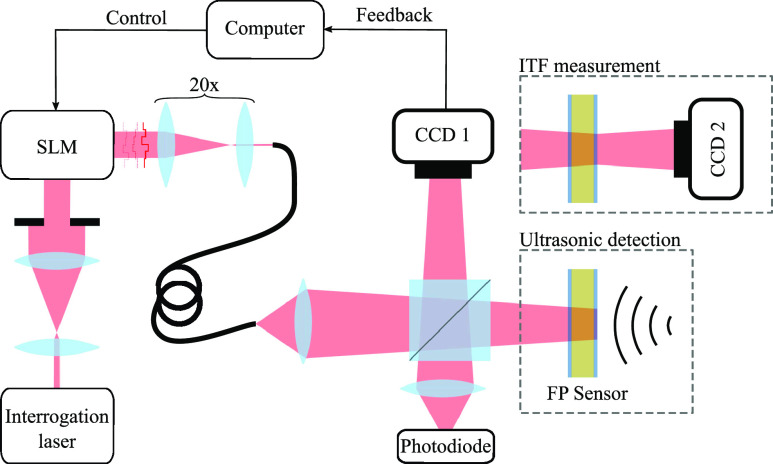
Schematic of an experimental system for mapping US field through MMF. Configurations used for ITF measurement and US detection are inset. The MMF used has an NA of 0.1 and a core diameter of 105 *μ*m, supporting ∼115 modes per polarization. On the FPUS plane, the illuminated region was a circle of 2.1 mm diameter and the speckle grain size was ∼155 *μ*m. The FPUS was similar to the one described in Zhang *et al.*,^1^ with a parylene-C^28^ spacer of 40 *μ*m thickness, and dielectric mirrors with a reflectivity of 98.8%.

To focus the light onto a particular position on the FPUS, a GA was used maximize the brightness at this position. To do so, first, a set of random phase patterns were displayed on the SLM. A subset of the phase patterns maximizing the brightness at the target location were selected and mixed to generate new phase patterns to display. This procedure was iterated, resulting in the gradual formation of a focus at the target region. To reduce the optimization time, the SLM elements were grouped into a smaller number of pixel groups, within which the same phase modulation was applied. To maximize the achievable “power ratio” (fractional power deliverable to the focus), the illuminated region on the SLM was chosen such that the number of illuminated pixel groups was equal to the number of modes supported by the MMF.

The procedure was used to focus light onto the FPUS at a location co-incident with the center of the camera's field of view. To examine the change to the field, images were recorded before and after WFS. The image obtained prior to WFS is plotted in Fig. 3(a). As expected, it contained seemingly random speckle. The image obtained after WFS is plotted in Fig. 3(b). Due to the WFS, it contains a focus in the intended location.

**FIG. 3.**
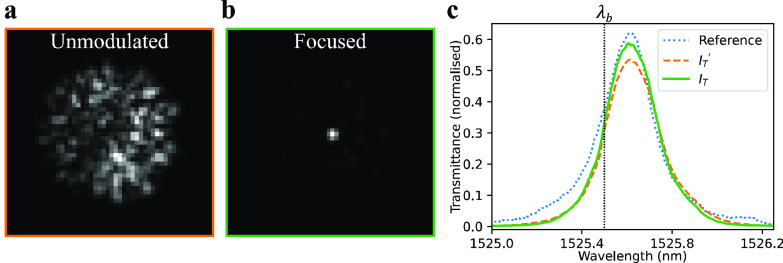
(a) Images of the beam profiles incident on the FPUS without SLM modulation and after a focus has been formed by WS. (b) ITFs recorded when the FPUS was illuminated by the unmodulated and focused field shown in (a) and in a traditional free-space scanner. The bias wavelength $\lambda_{b}$ is marked. The free space beam was a Gaussian beam with a ∼50 *μ*m beam waist.

To explore focusing at different locations and obtain an indication of the achievable power ratio, the above experiment was repeated in different conditions, including with different numbers of GA iterations, and different target locations for the focus. It was found that near-diffraction-limited foci could be produced anywhere in the field of view. The power ratio typically increased with the number of GA iterations, with a maximum value of ∼60% (after ≳50 iterations). The remainder of the optical power formed an effectively random low-intensity background speckle pattern.

After focusing the beam onto the FPUS, measuring ultrasound required determining an appropriate FPUS bias wavelength, $\lambda_{b}$ (Ref. 1), at the edge of a resonance, thus measuring the ITF. In conventional FPUS systems, spatially resolved ITFs are measured simply by scanning a Gaussian interrogation beam to points of interest on the FPUS, and measuring the reflectivity or transmissivity as a function of wavelength at each point. Here, however, the wavelength dependent nature of the modal dispersion of the MMF presents a challenge. Namely, any focus formed by WFS at one wavelength decays back into pseudo-random speckle if the wavelength is detuned away from the original wavelength. As such, practically speaking, accurately measuring the section of the ITF near to $\lambda_{b}$ with a spot formed via WFS required the WFS to be done using a wavelength near $\lambda_{b}$. This required the approximate bias wavelength to be known in advance.

To address this need, a four-step procedure was followed to obtain ITFs and bias wavelengths valid for spots produced via WFS. First, prior to WFS, an ITF, $I_{T}\prime \left(\lambda \right)$ was obtained under random illumination. Second, an estimated bias wavelength, $\lambda_{b}\prime$, was obtained by locating the wavelength of maximum derivative in $I_{T}\prime \left(\lambda \right)$. Third, the wavelength was tuned to $\lambda_{b}\prime$ and WFS applied to obtain an SLM pattern producing a focus at a chosen location. Finally, while projecting that same SLM pattern, the ITF measurement process was repeated, yielding the ITF, $I_{T}\left(\lambda \right)$ from which $\lambda_{b}$ could be extracted.

The above procedure was applied to measure an ITF and a bias wavelength at a chosen point on the FPUS. To measure the transmitted power, the camera was temporarily repositioned behind the FPUS. To measure $I_{T}\prime \left(\lambda \right),$ a flat phase pattern was displayed on the SLM, and the optical wavelength set to 1524.65 nm. The wavelength was then successively increased in steps of 4 pm, up to a maximum wavelength of 1526.65 nm, while the transmitted power was recorded. The recorded ITF was then normalized with respect to the total power incident on the FPUS, measured using the photodiode in the path of the reflected light. The resulting normalized ITF is plotted in Fig. 3(c), labeled $I_{T}\prime$. As expected, a single sharp resonance is evident, resulting in a peak in transmission at ∼1525.6 nm. After applying WFS to produce a focus on the FPUS at an approximate bias wavelength $\lambda_{b}\prime$, the ITF measurement process above was repeated to yield $I_{T}\left(\lambda \right),$ which is plotted in Fig. 3(c). $I_{T}\left(\lambda \right)$ was similar to the $I_{T}\prime \left(\lambda \right)$, indicating that the change in a beam structure has not significantly affected the ITF. To further verify that the FPUS was behaving as expected, an ITF was also measured using a conventional free space FP scanner^1^ and plotted in Fig. 3(c) labeled “reference.” This ITF is similar to that obtained via WFS, again indicating that differences in the beam structure did not significantly affect the FPUS's behavior. The lack of broadening in $I_{T}\prime \left(\lambda \right)$ relative to $I_{T}\left(\lambda \right)$ and the reference ITF additionally indicates that sensor is of sufficiently uniform thickness over the illuminated region to allow the same $\lambda_{b}$ to be used for any focus position in this area.

To demonstrate spatially localized ultrasound measurements, an experiment was devised that involved sampling a known spatially varying ultrasonic wavefront at four locations. A schematic of the setup is plotted in Fig. 4(a). Briefly, a pulsed US transducer delivered a plane wave at a known angle to the FPUS. In these conditions, all points on the FPUS encountered a time-varying ultrasound wave comprising a single pulse. However, due to the off-axis angle of the transducer, different points on the FPUS received the pulse at different times. Thus, measuring the arrival times of the US waves provided a means to verify their spatial localization.

**FIG. 4.**
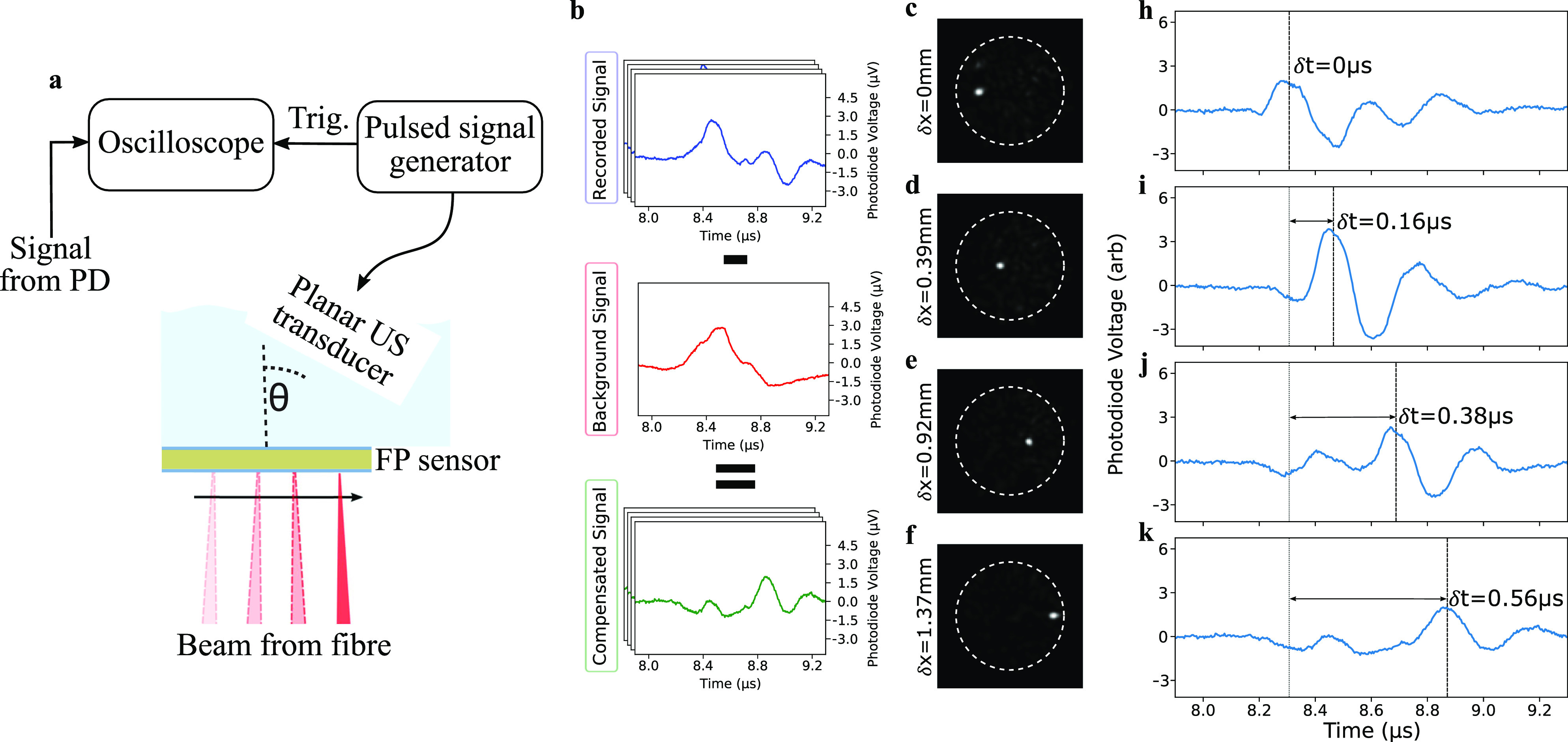
(a) Schematic of the experimental setup used to demonstrate spatial localization of US measurements. θ was set to 40°. (b) Illustration of the signal processing used for background subtraction. (c)–(f) Images of the four WS formed foci used to illuminate the FP sensor, alongside the corresponding recorded ultrasound time-traces (h)–(k). The speed of sound in the ultrasound coupling gel between the transducer and sensor was ∼1500 ms^−1^.

The setup was used to measure ultrasonic waveforms after focusing the beam onto the FPUS at an appropriate bias wavelength using the methods described above. The measurement points were chosen to be positioned along a line perpendicular to the axis of rotation of the transducer. In this arrangement, the difference in the times of arrival of the pulses expected at each point was δt=δx/(v sin θ), where θ was the angle, δx was the distance between the points, and *v* was the speed of sound in water. As a reference, a waveform was also captured using random speckle illumination.

One of the raw ultrasonic waveforms obtained using a WFS-formed optical spot is plotted in Fig. 4(b). This, along with the other WFS-based waveforms, contained two evident wave components. The first was unique to each measurement, and had the expected temporal shape given the known output wave characteristics of the transducer. The second was common to all the waveforms (as well as the reference measurement with random illumination) and was, thus, presumed to be due to uncontrolled “background” light, i.e., the residual remaining background speckle outside the WFS-formed foci. To suppress this background, a simple correction was performed. The process is illustrated in Fig. 4(b). Briefly, the reference waveform obtained under random illumination was subtracted from each of the other waveforms; under the assumption, this was a suitable approximation of the background.

The resulting background-subtracted waveforms are plotted in Figs. 4(h) and 4(k), alongside the corresponding illumination images showing the effectively “scanned” interrogation beam [Figs. 4(c) and 4(g)]. As expected, the waveforms contained a short pulse. Also as expected, the pulse arrived at different times, in agreement with the quantitative predictions based on the angle of the transducer and the distance from its axis of rotation. This agreement shows that the ultrasonic measurements were localized to the regions of the WFS-formed optical spots. The magnitude of the detected pulses varied between the waveforms, likely due to a combination of variability in the power ratio and spatial variation in the sensitivity of the FPUS. In addition, a residual background signal is still visible [clearest in Figs. 4(j) and 4(k)]. This may be due to the approximate nature of the background subtraction.

The presented experiments show that an FPUS can be interrogated through MMF, in turn allowing spatially localized US measurements. As well as this main finding, certain features of the experiments highlight directions for further research.

One feature of the experiments is that they were slow, but they could be sped up to enable high-resolution imaging. Specifically, focusing light onto the FPUS took tens of minutes, limiting the number of measurements, and reducing the practicality of imaging. The limiting factors were the pattern rate of the SLM (<10 Hz pattern rate) and the iterative nature of the GA. Both could be addressed. For instance, swapping the SLM for a faster digital-micromirror device (DMD)^18^ could provide a >10 kHz pattern rate. Meanwhile, the GA could be replaced with a TM measurement approach, allowing measuring the relationship between all the input and output modes of the MMF in an efficient pre-calibration step.^15^ This could significantly reduce the acquisition time, enabling imaging through a >1000 mode MMF in a matter of seconds.

A second feature of the experiments was that, while the detected US waves were partially localized to the WFS-formed interrogation spots, a background signal was also observed due to the uncontrolled portion of the speckle. This effectively resulted in “crosstalk” between the ∼115 measurement channels in the MMF, only four of which were used to make the actual measurements. Here, a simple approximate subtraction was used to partially suppress this crosstalk. However, more sophisticated approaches could be used to reduce it more completely. For example, if most of the available channels in the MMF were characterized by measuring the TM, then techniques analogous to those used in multiple-input multiple-output communications systems^19^ and single pixel imaging^20^ could be applied.

A final feature of the experiments was that the MMF was held stationary throughout. The purpose was to avoid any complications due to the TM of the MMF changing in response to environmentally induced fiber strain.^21^ This is relatively common practice in MMF imaging research and is also compatible with producing rigid imaging probes.^22^ However, extending the methodology for use in flexible probes will require adopting or developing a method of compensation to enable beam scanning robust to fiber bending.^21,23,24^

In summary, we showed that it is possible to spatially map US fields using an FPUS interrogated through MMF. In this proof-of-concept work, the number of detection points was low due to practical constraints. However, speeding up the system could allow tractable ultrasonic imaging with >1000 channels. Uncontrolled background light produced inter-channel crosstalk. However, this too could be mitigated, increasing the signal fidelity.

By providing a means to map ultrasonic fields through narrow, inexpensive, and lightweight fibers, this could lead to the development of a range of new ultrasonic and photoacoustic imaging systems. As for other devices based on MMF, such systems could benefit from compatibility with multi-modality imaging through a single fiber. This interrogation scheme may provide additional advantages to FPUS-based imaging, as control over the structure of the interrogation beam could lead to higher sensitivity^25,26^ or allow for angle-based sensor biasing.^27^ Resulting systems could include environmentally resilient industrial ultrasound probes capable of inspection through narrow openings, handheld photoacoustic or ultrasonic imaging systems, and clinical gastrointestinal or surgical endoscopes capable of imaging at otherwise hard-to-access sites in the human body.

## Data Availability

The data that support the findings of this study are available from the corresponding author upon reasonable request.
